# Designing combination therapies for cancer treatment: application of a mathematical framework combining CAR T-cell immunotherapy and targeted radionuclide therapy

**DOI:** 10.3389/fimmu.2024.1358478

**Published:** 2024-04-18

**Authors:** Vikram Adhikarla, Dennis Awuah, Enrico Caserta, Megan Minnix, Maxim Kuznetsov, Amrita Krishnan, Jefferey Y. C. Wong, John E. Shively, Xiuli Wang, Flavia Pichiorri, Russell C. Rockne

**Affiliations:** ^1^ Department of Computational and Quantitative Medicine, Beckman Research Institute, City of Hope National Medical Center, Duarte, CA, United States; ^2^ Department of Hematology and Hematopoietic Cell Transplantation, Beckman Research Institute, City of Hope National Medical Center, Duarte, CA, United States; ^3^ Department of Hematologic Malignancies Translational Science, Beckman Research Institute, City of Hope National Medical Center, Duarte, CA, United States; ^4^ Department of Molecular Imaging and Therapy, Beckman Research Institute, City of Hope National Medical Center, Duarte, CA, United States; ^5^ Department of Radiation Oncology, City of Hope National Medical Center, Duarte, CA, United States

**Keywords:** radionuclide, combination therapy, myeloma, CAR T cells, daratumumab, mathematical model, targeted alpha therapy

## Abstract

**Introduction:**

Cancer combination treatments involving immunotherapies with targeted radiation therapy are at the forefront of treating cancers. However, dosing and scheduling of these therapies pose a challenge. Mathematical models provide a unique way of optimizing these therapies.

**Methods:**

Using a preclinical model of multiple myeloma as an example, we demonstrate the capability of a mathematical model to combine these therapies to achieve maximum response, defined as delay in tumor growth. Data from mice studies with targeted radionuclide therapy (TRT) and chimeric antigen receptor (CAR)-T cell monotherapies and combinations with different intervals between them was used to calibrate mathematical model parameters. The dependence of progression-free survival (PFS), overall survival (OS), and the time to minimum tumor burden on dosing and scheduling was evaluated. Different dosing and scheduling schemes were evaluated to maximize the PFS and optimize timings of TRT and CAR-T cell therapies.

**Results:**

Therapy intervals that were too close or too far apart are shown to be detrimental to the therapeutic efficacy, as TRT too close to CAR-T cell therapy results in radiation related CAR-T cell killing while the therapies being too far apart result in tumor regrowth, negatively impacting tumor control and survival. We show that splitting a dose of TRT or CAR-T cells when administered in combination is advantageous only if the first therapy delivered can produce a significant benefit as a monotherapy.

**Discussion:**

Mathematical models are crucial tools for optimizing the delivery of cancer combination therapy regimens with application along the lines of achieving cure, maximizing survival or minimizing toxicity.

## 1 Introduction

Chemotherapy and external beam radiation therapy have been traditional approaches for treating hematological malignancies. External beam radiation therapy has typically been employed for treatment of solitary plasmacytomas and as a palliative measure for more widespread disease (1, 2). The primary disadvantage of external beam radiotherapy is the toxicity to normal cells present near malignant cells in the bone marrow. Thus, its role has been limited in the treatment of hematological malignancies. In contrast, immunotherapy-based approaches have been employed in standard regimens and have led to significant improvements in patient disease remission (3). The dysregulation of the immune system in multiple myeloma (MM) and its targeting by immunotherapies has been a key reason for immunotherapy success (4). In particular, Chimeric Antigen Receptor T cells (CAR-T cells) have recently come to the fore due to their efficacy against several hematological malignancies including MM, leukemia and B-cell malignancies (5). CAR-T cells are T cells that have been engineered to target a receptor on the tumor cells thus binding them to tumor cells for direct effect. B-Cell Maturation Antigen (BCMA) targeting CAR-T cells have recently been approved by the FDA for treatment of MM (6). While these novel immunotherapies have created a significant impact, most patients still experience relapse, leading to unsuccessful treatment (7), supporting the need to develop novel combinatorial approaches for complete disease eradication.

Targeted radionuclide therapy (TRT) is a form of radiation therapy in which a radionuclide delivering radiation is attached to an agent that targets tumor cells (8). The advantage of TRT is that it is both highly targeted and delivered systemically. In addition, the radionuclide can be chosen with a half-life that is appropriate for balancing efficacy and toxicity of the treatment. For example, we have shown that the targeted alpha particle therapy (TAT) with ^225^Ac conjugated to the CD38 receptor targeting antibody daratumumab demonstrated superior efficacy without added toxicity in treatment of disseminated multiple myeloma in a mouse model as compared to a beta particle emitter ^177^Lu (9). The shorter range (< 100 μm) but higher potency (given by their high linear energy transfer) of alpha particles emitted from ^225^Ac and its daughters was crucial in targeting the cancer cells but sparing the normal tissue cells in the bone marrow. While TAT was associated with increased survival, it alone did not result in curative responses. To address this limitation of radionuclide therapies, we and others (10–12) have investigated the addition of immune-based therapies to TRT as a potentially curative combination therapy approach.

Selecting the dosing, timing, and sequencing of any combination therapy approach experimentally is challenging due to the number of possible combinations to be tested; therefore a more efficient method of experimental design to achieve optimal therapy regimens is useful and increasingly becoming essential (13). Fortunately, mathematical models aided by experimental data exemplify a way for achieving optimal combination of therapies. Combining multiple therapies can result in synergistic, additive or antagonistic effects. Methods have been proposed to quantify (14, 15) and optimize (16–18) these effects. Combination therapy optimization using mathematical models can be performed at several levels depending on our knowledge of the system parameters (19). Many types of therapy combinations have been investigated using mathematical models (20). In particular, mathematical models of tumor-immune system dynamics have been proposed to optimize and personalize immunotherapies (21–23) either on their own or in combination with chemotherapy (24). Additionally, mathematical modeling of radiation therapy using the linear-quadratic model (25) to optimize patient-specific treatment regimens has been investigated for decades (26, 27). The proposed radiobiological models have been used to study both tumor and normal tissue radiation dose-response effects (28–30). The modeling efforts using radiobiological models and their variations span both external beam, brachytherapy as well as targeted radionuclide radiotherapies (30–35). Recognizing the synergy between immunotherapies and radiation therapy, mathematical models utilizing the external beam radiation therapy and immunotherapies have been proposed (36, 37) in order to tailor the dose of external beam radiation therapy to elicit systemic immune response as well as to study the effect of radiation therapy on different immune populations (38, 39).

We recently proposed a mathematical model for optimization of targeted radionuclide therapy with CAR-T cell therapy (40). The model considered the tumor response to targeted radionuclide and CAR-T cell therapies as monotherapies and preclinical data from each set of monotherapy experiments was used to characterize the model. Different timing schedules of TAT and CAR-T cell therapies were tested *in silico*, and it was shown that the timing between the two therapies for maximizing the survival metrics was highly dependent on the tumor proliferation rate. Elucidating the mathematical parameters relevant for multiple therapies gives a novel way of investigating the dosing, timing, and the sequence of combination therapies and generate *in silico* therapeutic regimens that can then be tested *in vivo*.

Here we validate the mathematical model against experimental data where the timing of the CAR-T cell therapy is varied keeping the TAT dosage and timing constant. Model parameters are elucidated from the experimental data to optimize the timing of CAR-T cell therapy post-TAT by maximizing the progression-free survival. The effect of fractionated dosing of both TRT and CAR-T cell therapies on the survival metrics is also studied. In addition, multiple dosing strategies for TRT and CAR-T cell therapies are tested to analyze whether the splitting and scheduling of the doses result in improved tumor control or survival.

## 2 Methods

### 2.1 Mathematical model

**Figure 1:**
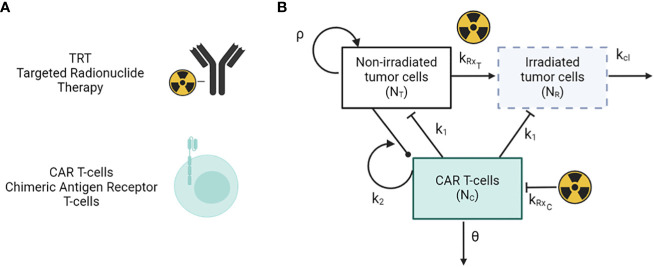
**(A)** Antibody-based TRT and CAR T-cells therapy modalities. **(B)** Schematic of the mathematical model of TRT and CAR-T cell therapy.


The framework of the combined mathematical model for TRT and CAR T-cell therapy dynamics is given by the set of differential equations as described below (**Figure 1**) (40). This simplified model considers only the tumor cells, CAR-T cells, and action of the TRT.

(1) $$\frac{dN_{T}}{dt}=\rho N_{T}-H\left(t-\tau_{TRT}\right)k_{Rx_{T}}N_{T}-H\left(t-\tau_{CAR\;T}\right)k_{1}N_{T}N_{C}$$

(2) $$\frac{dN_{R}}{dt}=\;H\left(t-\tau_{TRT}\right)k_{Rx_{T}}N_{T}-H\left(t-\tau_{CAR\;T}\right)k_{1}N_{R}N_{C}-k_{cl}N_{R}$$

(3) $$\frac{dN_{C}}{dt}=k_{2}\left(N_{T}+N_{R}\right)N_{C}-H\left(t-\tau_{TRT}\right)k_{Rx_{C}}N_{C}-\theta N_{C}$$

(4) $$k_{Rx_{i}}=\;\alpha_{i}R_{0}e^{-\lambda \left(t-t_{TRT}\right)}+\frac{2\beta_{i}R_{0}^{2}}{\gamma -\lambda }\left(e^{-2\lambda \left(t-t_{TRT}\right)}-e^{-\left(\lambda +\gamma \right)\left(t-t_{TRT}\right)}\right)\gamma \lambda$$

Here, N_T_ and N_R_ represent the number of tumor cells that are unirradiated and irradiated respectively. N_C_ represents the number of CAR-T cells in the system, and k_1_ and k_2_ represent the killing rate of tumor cells by CAR-T cells and the proliferation/exhaustion rate of CAR-T cells respectively. θ and k_cl_ are the clearance rates of CAR-T cells and irradiated tumor cells from the system. t_TRT_ and t_CART_ are the time points at which TRT and CAR-T therapy are given via a Heaviside function H(t). The parameters α and β are radiobiological constants from the linear-quadratic model with β = 0 for high linear energy transfer alpha particle-based therapy. The parameter R_0_ is the initial dose rate given as R_0_ = η A_inj_, where A_inj_ is the injected radioactivity. Parameters, values, and units are given in **Table 1**. The immune system as well as other populations of normal cells are not considered in this simplified model. The subscript *i* in equation (4) indicates the type of cell population which can either be T for tumor or C for CAR-T cells.

**Table 1:** Constants and global parameters for the mathematical model estimated from experimental data.

Parameter	Symbol	Value	Comments
Effective decay constant (1/day)	λ	0.07	Accounts for biological clearance and physical decay
Tumor proliferation rate (1/day)	ρ	0.208	Global parameter set optimized value
Clearance rate of irradiated tumor cells (1/day)	k_cl_	0.45	Global parameter set optimized value
CAR-T cell killing rate (1/day/cell)	k_1_	3.01x10^-7^	Optimized from data
CAR-T cell proliferation/ exhaustion rate (1/day/cell)	k_2_	2.34 × 10^−14^	Global optimized and kept as constant for individual mice
CAR-T cell death rate (1/day)	θ	0.035	Global parameter set optimized value
Tumor cell radiosensitivity (1/Gy) *	α_T_	1.43	Global parameter set optimized value
CAR-T cell radiosensitivity (1/Gy) *	α_C_	1.01	Global optimized and kept as constant for individual mice
Activity to dose conversion factor (Gy/day/μCi)	η	1.44	Global optimized and kept as constant for individual mice

*Note that the radiosensitivity coefficients incorporate the effect of the radiobiological effectiveness of high linear-energy transfer radiation as is the case in ^225^Ac alpha particle therapy.

### 2.2 Experimental data

To benchmark the model and to optimize the timing of CAR-T and TRT therapies, data on monotherapies of TRT using ^225^Ac-daratumumab targeting CD38 receptor and CAR-T cells targeting CS1 receptor as well as combination of these therapies was analyzed (11). Briefly, NOD.Cg-Prkdcscid Il2rgtm1Wjl/SzJ mice (NSG; 6–10 weeks old; Jackson Laboratory) (IACUC 21034) were engrafted with 5 × 10^6^ MM.1S eGFP-ffluc lines intravenously (I.V.) and randomized into groups 6 days post tumor injection (day 6), based on bioluminescence imaging (BLI). All mice were followed weekly over the course of therapy using BLI to measure tumor burden. Day 0 was taken as the day MM.1S cells were inoculated in the mice. Six groups of mice (n = 8 each except CAR-T only group) with multiple myeloma are considered: *(a)* Untreated mice serving as controls (Group-0) *(b)* TRT (day 7) only post tumor inoculation (Group-T7) *(c)* TRT (day 7) and CAR-T cells (day 18) (Group-T7C18) *(d)* TRT(d7) and CAR-T cells (d25) (Group-T7C25) *(e)* TRT (d7) + CAR-T (d32) (Group-T7C32) and *(f)* CAR-T cell monotherapy administered on day 7 (n = 7) (Group-C7). For groups c, d, and e, the CAR-T cell doses were planned to be administered on day 14, 21, and 28 respectively. However, due to experimental logistical considerations, the actual dates of administration were 18, 25, and 32 respectively. BLI images and raw data can be found in the experimental publication (11).

### 2.3 Benchmarking model parameters

The tumor proliferation rate ρ was calculated from untreated control tumor data by fitting an exponential function to the individual mice tumor burden trajectories over time as measured with BLI (**Supplementary Figure 1**). The average BLI measurement on day 7 post inoculation and the average proliferation rate ρ across all untreated mice was then used to back calculate what the BLI would have been on day 0 based on the exponential tumor growth formalism. A single BLI to tumor cell conversion factor was then calculated by taking the ratio of BLI flux on day 0 and the number of injected MM.1S cells. For the treated groups, the following parameters and quantities were held constant based on the experimental conditions: CAR-T cell dose (N_C0_), injected radioactivity (A_0_), effective TRT decay rate (λ), and initial tumor burden (N_T_(t = 0)). Other parameters (ρ, η, α_T_, α_C,_ k_1_, k_2_, θ and k_cl_) were allowed to vary and were optimized simultaneously for all cohorts yielding a single parameter set for all mice. This approach identifies parameters for CAR-T cell and tumor cell radiosensitivity, tumor proliferation, CAR-T cell and TRT killing rate and TRT clearance that are specific to the MM.1S myeloma cell line, the CS1 CAR T-cells and the ^225^Ac-daratumamab therapies across treatment groups. The parameter set that is common and shared across treatment groups for all mice is referred to henceforth as the global parameter set.

Once the global parameter set for TRT and CAR-T cells therapy was calculated as above, these parameter values were set as initial conditions for individual mice to allow for individual mice variations across the parameter set. Thus, mice-specific parameters were calculated by allowing the individual parameters to vary by +/- 50% from the global parameter values. Mice-specific parameters included: ρ, α_T_, k_1_, θ and k_cl_. For individual mice data optimization, η, α_C_, and k_2_ were held constant to the global parameter set.

### 2.4 Optimizing single administration of CAR-T therapy with respect to TRT and influence of therapy interval on model parameters

The benchmarked global parameter set was used to test the optimal timing of CAR-T cell therapy post TRT. With TRT injection on day 7 after MM.1S cell inoculation, the time of CAR-T cell delivery was varied from day 8 (following day administration) until day 50 (43 days following TRT). Progression free survival (PFS) was calculated for each scenario. Progression-free survival was defined as the amount of time from the start of TRT for the tumor burden to attain the same size as at the start of treatment. If no reduction in tumor burden was observed, the PFS was set to zero days. CAR-T cell therapy timing that maximized the PFS was taken as the optimal therapy timing.

The influence of variation in individual model parameters on the optimal timing of CAR-T cell therapy after TRT was tested. For this purpose, the parameter set obtained from fitting the model to individual mice tumor trajectories (with 50% uncertainty from global parameter set) was used. The minimum and maximum values of the individual parameters were calculated from this parameter set and a new synthetic parameter set was created with parameters randomly and uniformly generated between these limits. Using this synthetic parameter set, the range of CAR-T injection days that resulted in the maximum PFS was calculated. In this manner, 1000 synthetic parameter sets were generated by sampling the parameters 1000 times. The resulting histogram of CAR-T injection days for maximum PFS was generated by summarizing the data from all 1000 simulations. For visualization purposes, the dependence of PFS on TRT to CAR-T therapy interval for five synthetic parameter set runs is shown and a histogram of CAR-T infusion days that maximized the PFS for each of these 5 parameter sets is shown. Similar analysis was done for parametric variation with 10% and 30% uncertainty (**Supplementary Figure 3**).

### 2.5 Impact of TRT and CAR-T cell therapy dosing and scheduling on survival metrics

Both TRT and CAR-T cell doses were varied between 20% of the experimental dose to 200% of the experimental dose. For TRT this range was 1.48 to 14.8 kBq and for CAR-T cells it was from 0.2 to 2 million cells. Both therapies were simulated with the model as monotherapies *in silico* to evaluate the impact of therapy dose on the time to minimum tumor burden (t_min_), the progression free survival and the overall survival (OS). Overall survival was defined to be the time interval between the tumor cell injection and the time at which the number of tumor cells reaches 10^11^. The minimum dose demonstrating an advantage in PFS was noted. Time to minimum tumor burden (t_min_) is defined as time difference between the start of the first treatment and the day of the minimum tumor burden. If no reduction in tumor was observed t_min_ was set to zero. The impact of two doses of TRT or two doses of CAR-T therapy were tested. Mathematically, this is done by adding the second TRT or CAR-T therapy term to Equations (1) through Equation (4). For TRT, two doses of 3.7 kBq each were simulated with variable timing between them. For the CAR-T cell therapy, two doses of 0.5 million cells with variable timing between them were simulated. Minimum therapy interval demonstrating an advantage in PFS was noted.

### 2.6 Optimizing multiple administrations of CAR-T and TRT treatments

Based on the evaluation of tumor burden at different CAR-T and TRT dose levels, a multiple administration dosing scheme was created. A single dose of 7.4 kBq of TRT was administered for maximum efficacy while two separate doses of 1 million CAR-T cells each were administered. The therapies were administered *in silico* in an alternating fashion with the TRT dose given in between two CAR-T doses. While other regimens can also be explored, we tested this dosing scheme here.

Based on the above dosing schemes, a strategy for maximizing PFS was tested. First, CAR-T cell therapy (1 million cells – dose 1) was given on day 7. The timing of sequential TRT (7.4 kBq) and the subsequent CAR-T cell therapy (1 million cells – dose 2) was varied. Tumor burden, PFS and OS were calculated for each of the simulated therapeutic scenarios. To test the effect of model parameter variability on the optimal timing of the doses, the parameter limits from individual mice fits with 30% variation from baseline parameters are used to randomly generate 100 synthetic parameter sets, which was observed to sufficiently characterize the distribution. For visualization, two synthetic parameter sets are simulated and creation of histograms demonstrating the range of TRT and CAR-T therapy timings that yielded the highest PFS are shown.

## 3 Results

### 3.1 Benchmarking model parameter set for individual mice

**Figure 2:**
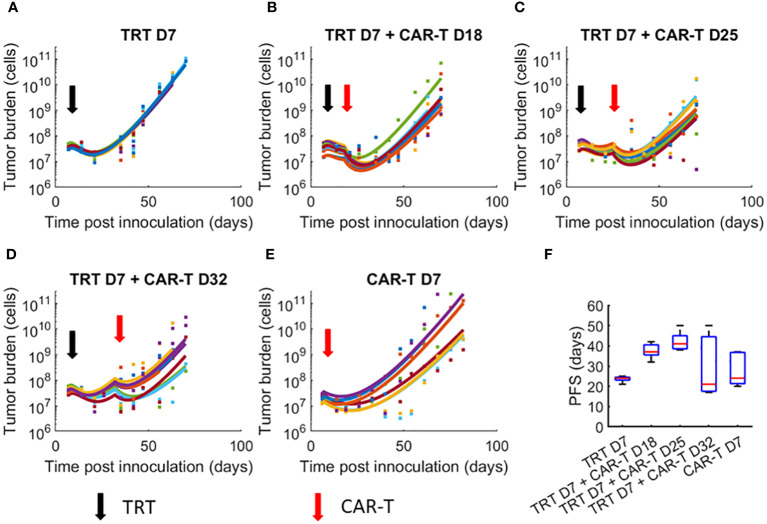
Dynamics of tumor growth in response to TRT and CAR-T combination therapies. Datapoints show the tumor cell numbers calculated from the experimental BLI data while the curves show the model fits to data. Individual mice parameters were limited to ±50% of the parameters obtained from the global parameter set. **(A)** TRT D7 only **(B)** TRT D7 + CAR-T cell D18 therapy. **(C)** TRT D7 + CAR-T cell D25 therapy. **(D)** TRT D7 + CAR-T cell D32 therapy. **(E)** CAR-T cell D7 therapy. **(F)** PFS obtained for each treatment group. The TRT D7 + CAR-T cells D25 therapy group demonstrated the highest PFS.

Due to the variability in response among mice within a treatment group, we fit the model to each mouse tumor growth curve, allowing 50% variation in the parameters from the global parameter set (**Figures 2A–E**). PFS for each group (**Figure 2F**) show that treatment group with TRT dose on day 7 and CAR-T cell dose on day 25 resulted in the greatest PFS. Large uncertainty in PFS was found for treatment with TRT on day 7 and CAR-T cell therapy on day 32, suggesting that the overall efficacy of the combination therapy decreases and becomes highly uncertain if the interval between therapies is too long. Analogous to **Figure 2**, the model-data fit using global parameters is provided in the supplement (**Supplementary Figure 2**).

### 3.2 Optimizing single administration of CAR-T therapy with respect to TRT and influence of therapy interval on model parameters

**Figure 3:**
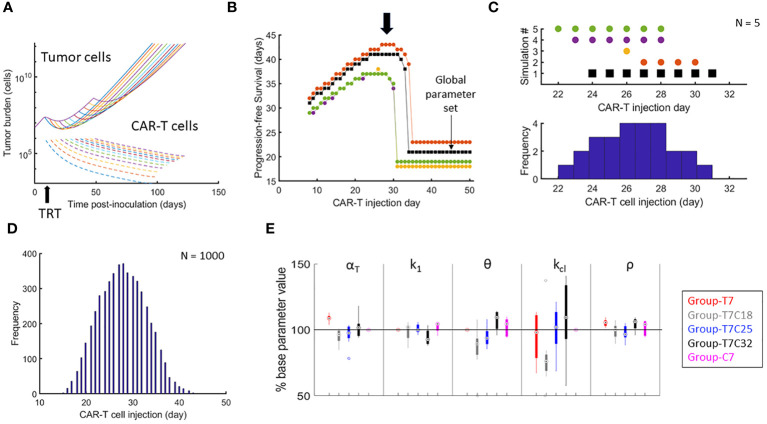
Optimizing the timing of CAR-T cell therapy after TRT and parametric variations attributed to treatment intervals. **(A)** Temporal development of tumor and CAR-T cells based on the global parameter set with TRT administered on day 7 and CAR-T cell therapy administered on one of the days from day 8 to day 50. **(B)** PFS calculated from day of TRT. Results of 5 synthetic parameter set runs are shown with global parameter set in black. For the global parameter set, maximum PFS is observed when CAR-T cells are delivered between day 25 and 34. Note that there is a range of CAR-T cell administration dates that yield highest PFS (black arrow). **(C)** The day when the PFS was maximum for each simulation run from B is shown along with the corresponding histogram demonstrating how the variability in parameters between different simulation runs impacts the variability in day of optimal CAR-T cell infusion. **(D)** Histogram showing the range of CAR-T injection days for which the maximum PFS was found with 1000 synthetic parameter set simulations. CAR-T cells infused on day 27 on average had the highest probability of maximizing PFS. **(E)** Distributions of model parameters for individual mice tumor growth curves are shown with parameters limited to ±50% of the parameters obtained from the benchmarked parameter set for (1) d7 TRT only (2) d7 TRT + d18 CAR-T cell therapy. (3) d7 TRT + d25 CAR-T cell therapy. (4) d7 TRT + d32 CAR-T cell therapy. (5) CAR-T cell monotherapy.

The global parameter set was used to simulate variable CAR-T therapy intervals post-TRT. Temporal dynamics of tumor and CAR-T cell numbers are shown in **Figure 3A**. PFS curves from 5 synthetic parameter sets (including the benchmarked one in black) are shown in **Figure 3B**. There is a range of therapy intervals for which the PFS is maximum (black arrow). The CAR-T cell administration day that yielded the maximum PFS for each of these curves is plotted (**Figure 3C**). The procedure for generation of histogram of optimal CAR-T cell therapy administration day for maximizing PFS using these 5 synthetic datasets is also shown. The histogram created from 1000 synthetic parameter sets shows a well-defined peak at day 27-28 post-tumor inoculation (**Figure 3D**) indicating this to be the CAR-T cell therapy administration day for maximizing PFS. The synthetic parameter sets used for the results shown incorporate 50% variability from the global parameter set. Similar results are obtained with 10% or 30% uncertainty in parameters (**Supplementary Figure 3**).

**Figure 3E** shows the parameter variation between groups. α_T_ is seen to be increasing with increased interval between therapies – indicating that more of the tumor reduction is due to TRT rather than CAR-T. Thus, the group-specific parameters show that the effectiveness of CAR-T therapy is lower with increased intervals between the therapy. Similarly, the value of k_1_ (CAR-T cell killing rate) is lower and θ (CAR-T cell persistence) is higher indicating again the reduced effectiveness of CAR-T cell therapy. These results can potentially point to a changing landscape of tumor cell mutations with increased tumor burden. This reduced effectiveness of CAR-T cell therapy with increased therapy interval is not captured with the overall survival metric obtained from the model.

### 3.3 Impact of TRT and CAR-T cell therapy dosing and scheduling on survival metrics

**Figure 4:**
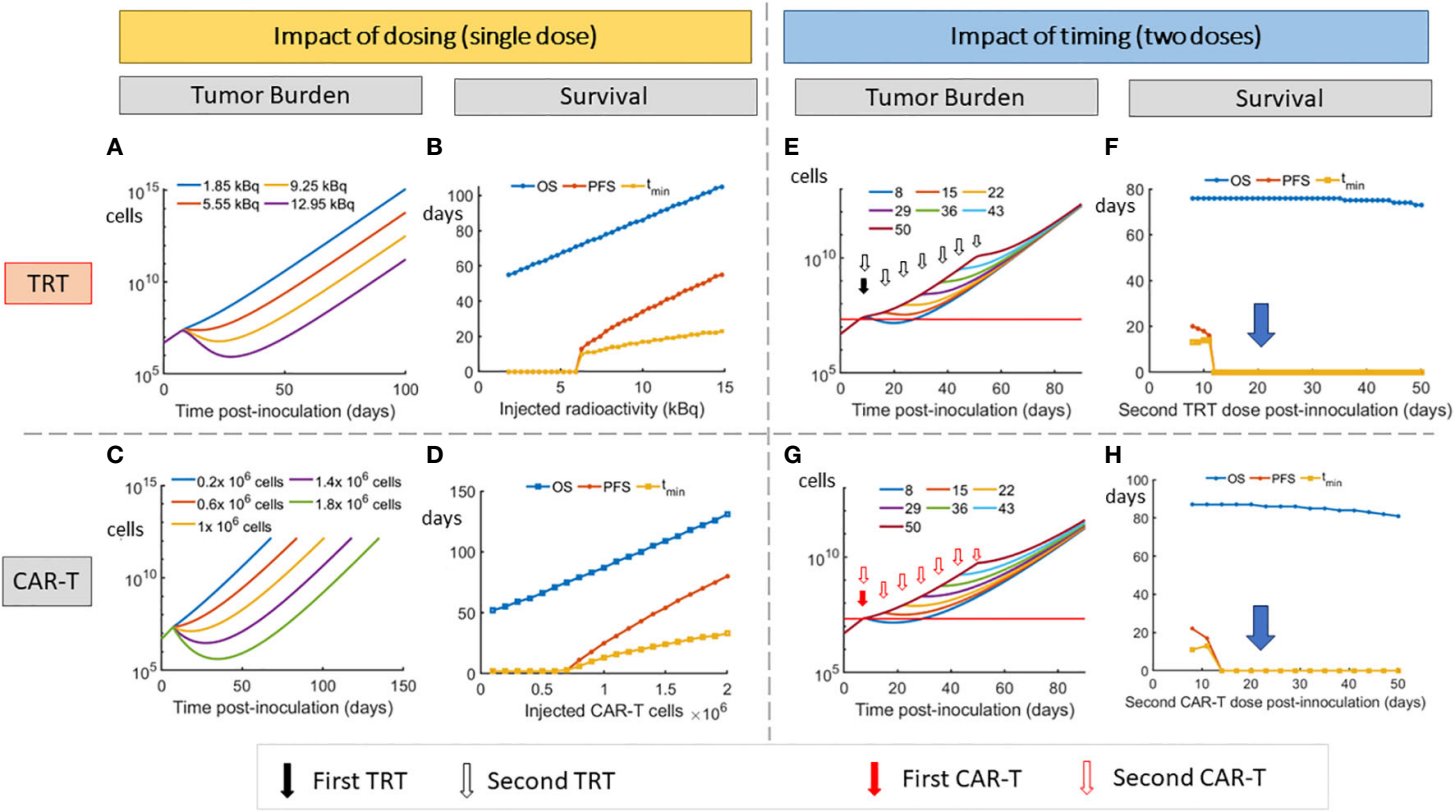
Impact of dosing and scheduling of TRT and CAR-T cell therapies on tumor burden and survival. Impact of TRT dose on **(A)** tumor volume and **(B)** Survival metrics. Impact of CAR-T cell therapy dose on **(C)** tumor volume and **(D)** Survival metrics. A threshold of approximately 6.3 kBq for TRT and 0.7 million CAR-T cells is required to observe an increase in PFS. Impact of timing of second dose of TRT on tumor burden **(E)** and survival **(F)**. Impact of timing of second dose of CAR-T cell therapy on tumor burden **(G)** and Survival **(H)**. When splitting the doses, the TRT dose was 3.7 kBq while CAR-T cell dose was 0.5 million cells for each administration. At these doses, the second dose needs to be given before day 12 for either TRT or CAR-T therapy to note any advantage in PFS. The model predicts that 7.4 kBq single dose or 1 million CAR-T cell dose that are split into two doses have a minimal effect on survival (D,E blue arrow).

**Figures 4A, B** show the impact of reduction in injected radioactivity on tumor burden and survival metrics. **Figures 4C, D** show the analogous impact of dose reduction in CAR-T cell numbers. In both cases, no reduction in PFS is observed below a certain dose level (about 6.3 kBq for TRT and 0.7 million cells for CAR-T cells). Doses below this level only result in slowing down of the disease burden. Overall survival is seen to linearly increase with increasing dose indicating the dose proportional gain in survival time.

Reducing the TRT dose (**Figure 4F**) of the first cycle to 3.7 kBq does not give an advantage in the PFS indicating that a critical dose is required initially for a multiple dose cycle. The highest PFS is found when the entire 7.4 kBq dose is delivered on day 7. Thus the 7.4 kBq dose that has been used in the experimental group is used for further simulations and the TRT dose is not split into two. Similar effects were observed when a 1 million CAR-T cell dose is split into two.

The impact of splitting TRT and CAR-T doses and varying the interval between these doses is shown (**Figures 4E–H**). At 3.7 kBq per TRT dose and 0.5 million cells per CAR-T dose, the second dose does not show improved PFS if it is administered after 5 days (after day 12) after the first dose (on day 7). The dependence of overall survival on therapy interval is seen to be low, with OS comparable across different therapy timing. However, this metric does not capture the parametric changes that happen when therapy interval is decreased (**Figure 4D**).

### 3.4 Optimizing multiple administrations of CAR-T and TRT treatments

**Figure 5:**
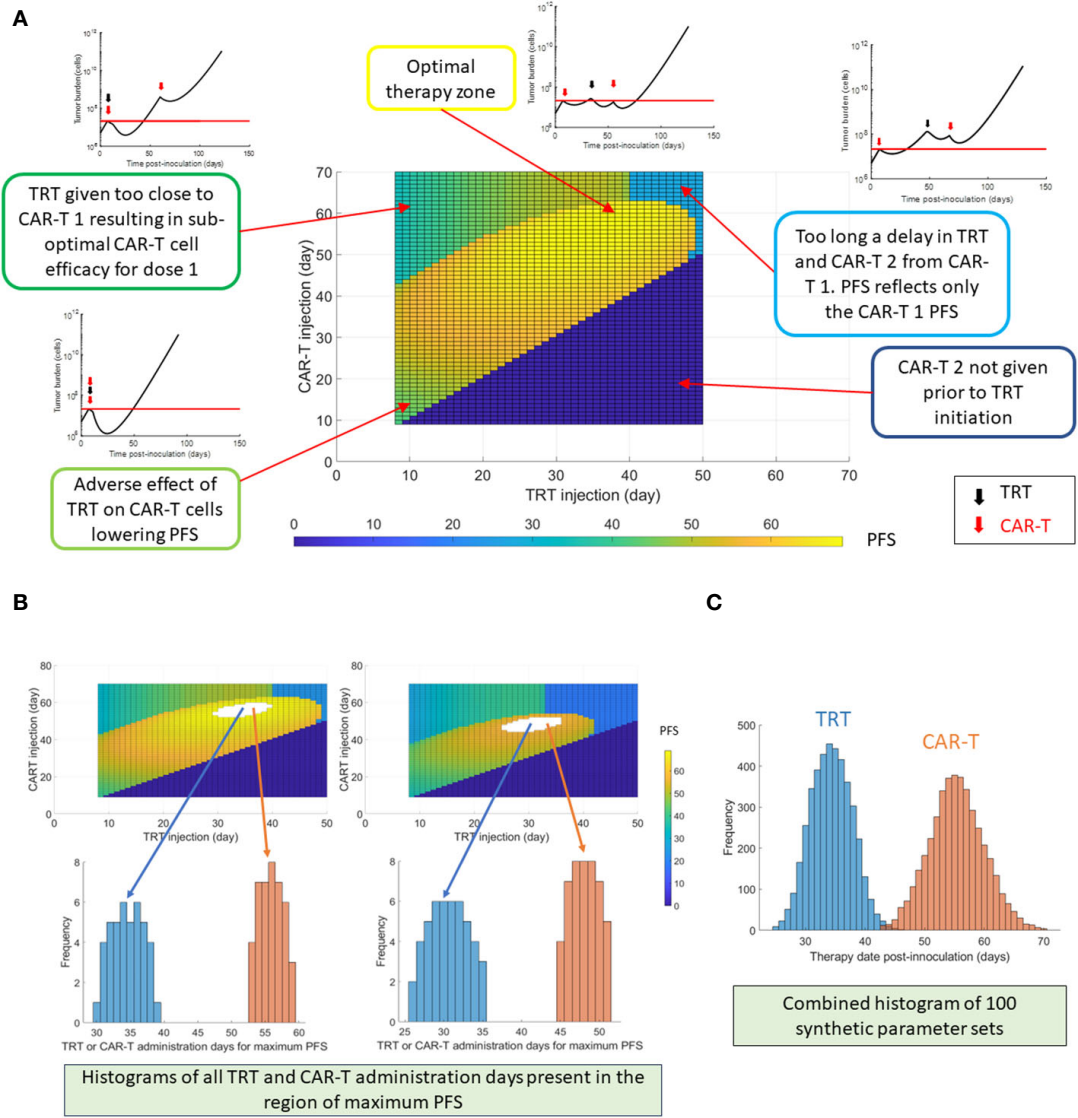
Optimization of multiple dosing schedules. The timing of the following sequential therapies was optimized: CAR-T cell therapy (dose 1 always on day 7) + TRT + CAR-T cell therapy repeated dose (dose 2), by maximizing the PFS. **(A)** Progression-free survival (PFS) shown as a function of TRT and CAR-T dose timings following the first CAR-T dose given on day 7. The tumor burden curves corresponding to different zones of the PFS map are also shown. **(B)** Example histogram creation of TRT and CAR-T cell dose timings that yielded the highest PFS based on two synthetic parameter sets. It should be noted that combination of both TRT and CAR-T therapy timings contribute to maximizing the PFS. The TRT and CAR-T histograms cannot be viewed in isolation. **(C)** TRT and CAR-T histograms created using 100 synthetic parameter sets generated within 30% uncertainty from global parameter set. Of these 100 synthetic parameter set simulations, maximum PFS was found roughly 400 times when the TRT injection was delivered on day 33 and CAR-T cells were infused on day 56. For each parameter set simulation, a PFS map as in **(A)** is observed – thus there are multiple CAR-T and TRT administration days that yield highest PFS.

After the first dose of CAR-T cell therapy, the timing of the following dose of TRT and CAR-T cell therapy needs to be optimized to yield maximum PFS (**Figure 5A**). If any of the therapies are given too close to each other, the resulting death of CAR-T cells due to radiation results in lower PFS. Similarly, having the therapies spaced too far apart can also result in lower PFS due to loss of benefit of the combination. The maximum PFS is observed when timing the second therapy close to the point when the tumor volume returns to the baseline. The creation of histograms for TRT and CAR-T therapies administration days that yielded the maximum PFS is shown (**Figure 5B**) using two synthetic parameter sets as examples. The procedure is analogous to the histogram creation in **Figure 3C** except that here there are two therapy sequence timings to be optimized (TRT and CAR-T dose 2) instead of one. Based on 100 synthetic parameter set simulations (**Figure 5C**) the maximum PFS is seen when TRT is delivered on day 33 and dose 2 of CAR-T cell therapy is given on day 56.

## 4 Discussion

In this work we show a modeling framework of combined TRT and CAR-T cell therapy applied to experimental data. We have elucidated the parameters that are relevant for this combination in a murine model of multiple myeloma. We show that splitting of therapy doses is advantageous only if the first therapy can produce a significant benefit on its own. The model provides a quantitative framework to optimize the dosing of immunotherapies and targeted radionuclide therapies.

We optimized the TAT and CAR-T model parameters such that a single parameter set with 50% uncertainty can explain the differences in the tumor burden curves between individual mice. Such an approach can facilitate *in silico* optimization of the two therapies, where a single parameter set is associated with each of the therapies. While the introduction of uncertainties in the parameters results in better individual tumor curve fits, more information about CAR-T cell dynamics within the system can be highly informative regarding the model parameters. In particular, the value of CAR-T cell proliferation (k_2_) was predicted to be small relative to other rate constants.

The optimized value of tumor proliferation rate (ρ = 0.21 day^-1^) is smaller than the one calculated from untreated controls (ρ = 0.27 day^-1^) which is used for BLI to tumor cell number conversion. There can be two reasons for this. First, the long-term growth of the tumor burden could be sigmoidal in nature instead of exponential as assumed in the model resulting in slowing down of the tumor growth at later stages as compared to the initial stages. Secondly, it could be that the post-treatment phenotype of the tumor cells could be more resistant and could grow slower in comparison to the pre-treatment phenotype. Such a scenario would also support the reduced effectiveness of CAR-T cell therapy when the interval between the therapies is increased (**Figure 3E**). The resistance of tumor cell population to therapy can be mitigated by combination therapy approaches targeting two different types of treatment sensitivities. Gatenby et al. (41) introduced the ecological evolutionary dynamics concept of ‘first strike-second strike’ approach (42) to strategically sequence therapies for tumor cure. The first strike reduces the size and heterogeneity of the tumor cell population while the second strike(s) pushes the population below a critical threshold that eventually results in tumor cell eradication. Relevant to the combinations used in this work, radiation therapy could be considered the tumor debulking treatment prior to CAR-T cell therapy (43).

Based on the mathematical model, it might be feasible to delay the delivery of the CAR-T cell dose post-TRT (CAR-T cells on day 25 *vs* 32), since the overall survival might increase. However, it comes at the cost of higher uncertainty in the PFS indicating that other factors that are not captured in the model might influence tumor growth that might let it escape treatment. Notably, the parameter evaluation for TRT + d32 CAR-T cell treatment indicates lower efficacy of CAR-T cells compared to other treatment combinations. This increased therapy interval can also potentially increase the mutational burden of the tumor and might lead to reduced therapy effectiveness.

In the current work, the optimization of multiple therapy combinations is done with the goal of maximizing PFS. Another strategy could be minimizing the tumor burden with the goal of curative response. For different therapy regimens, it could be tested whether tumor burden predicted by the model would be low enough for cure in contrast to a strategy for maximizing PFS, thus giving the model the flexibility to test different scenarios.

The mice experiments used in this work have been performed on immunocompromised mice. Thus, the effect of radiation on stimulating immune system is not present in this mouse model. This data presents a cleaner model system for elucidating the effect of CAR-T cells on the tumor volume by eradicating the influence of other immune system components that can affect tumor volume. The impact of radiation on the immune system will need to be incorporated for optimizing therapeutic regimens in humans.

Biological variability in both preclinical and clinical realms is a determinant of heterogeneous response to therapies both in terms of efficacy and toxicity. To this end, the model assumes that a set of model parameters is shared between the mice in the same group, albeit with some uncertainty. Here we have chosen the model parameters within a group to vary by 50% from the group mean. This uncertainty level was chosen to capture expected variation in mice groups based on our previous experience (40); and is enough to demonstrate the differences in mean response to therapy between the groups while at the same time accommodating qualitatively reasonable fits to individual mice. In patients, the model can be personalized better by delivering multiple cycles of therapy and evaluating the patient response to individual doses to tailor the next dose or therapy sequence. To this end clinical trials evaluating targeted radionuclide therapy using ^225^Ac-DOTA-Dara (Clinical trial identifier: NCT05363111) and CS1-CAR-T cell therapy (Clinical trial identifier: NCT03710421) as monotherapies in multiple myeloma patients are being evaluated to study patient response (toxicity and efficacy) to these agents. The combination of these therapies can bring synergistic effects and unique challenges. Radiation from TRT can give rise to non-specific immune response of CAR-T cells and might result in T-cell activation and priming. Abscopal effects of radiation that might be based on systemic responses can also result in increased efficacy compared to model predictions. A possible side effect especially relevant to targeted alpha particle therapies like ^225^Ac is the impact of these agents on the tumor vasculature. Depending on the specific tumor, its vasculature and the radiation dose delivered, a complex non-linear relationship might exist for vasculature killing that might impact the delivery of subsequent therapies. Studying the spatio-temporal nature of tumor response to different doses while at the same time imaging the accumulation of these agents in tumors (as being done in the ^225^Ac-Dara trial NCT05363111) can be key to develop more sophisticated mathematical models that can further optimize therapeutic regimen with targeted radionuclide therapies.

An obvious result is that higher dose of a therapy, the higher chance of cure; suggesting that the shorter the interval between two cycles of a therapy, the better the tumor reduction to the limit that CAR-T cells are not significantly damaged by the radiation. However, the interval between therapies cannot be shortened indefinitely. Reduced interval between TRT and CAR-T cell therapy results in death of CAR-T cells. On the other hand, reduced interval between two CAR-T cell doses or two TRT doses can be equivalent to a single high dose of that therapy. The radiation dose to organs at risk needs to be considered in a way that the rejuvenation of the organ at risk yields a therapeutic advantage when delivering a second cycle of the therapy. In the case of TAT, it could be the bone marrow while CAR-T cell toxicity can be in the form of cytokine release syndrome. Thus, a combination therapy where the two toxicity risks are unrelated to each other is seen to provide the best tumor control and delay tumor growth. Thus, the rationale for use of immunotherapies with TRT as part of a therapy regimen is strong and justifies its use for further investigation.

## Data availability statement

The data generated in this work are available in the article and the **Supplemental File**. The BLI data associated with the work is provided as another supplementary file. The associated code for analysis can be found at https://github.com/cohmathonc/TRT-and-CAR-T-cell-combination-therapy.git.

## Ethics statement

The animal study was approved by City of Hope Institutional Animal Care and Use Committee. The study was conducted in accordance with the local legislation and institutional requirements.

## Author contributions

VA: Conceptualization, Formal analysis, Investigation, Methodology, Validation, Writing – original draft, Writing – review & editing. DA: Data curation, Formal analysis, Investigation, Validation, Writing – review & editing. EC: Data curation, Formal analysis, Investigation, Validation, Writing – review & editing. MM: Data curation, Formal analysis, Investigation, Validation, Writing – review & editing. MK: Formal analysis, Validation, Writing – review & editing. AK: Formal analysis, Validation, Writing – review & editing. JW: Formal analysis, Validation, Writing – review & editing. JS: Formal analysis, Funding acquisition, Investigation, Project administration, Supervision, Validation, Writing – review & editing. XW: Formal analysis, Funding acquisition, Investigation, Project administration, Resources, Supervision, Validation, Writing – review & editing. FP: Formal analysis, Funding acquisition, Investigation, Project administration, Supervision, Validation, Writing – review & editing. RR: Conceptualization, Formal analysis, Funding acquisition, Investigation, Methodology, Project administration, Supervision, Validation, Writing – original draft, Writing – review & editing.
