# Supplementary material for: Designing combination therapies for cancer treatment: application of a mathematical framework combining CAR T-cell immunotherapy and targeted radionuclide therapy
Source: Front Immunol. 2024 Apr 18;15:1358478. doi: 10.3389/fimmu.2024.1358478 (PMC11063284; doi:10.3389/fimmu.2024.1358478)
Supplement: Supplementary file 1 [file DataSheet_1.docx]

**Supplemental Data**

*Estimation of tumor cell proliferation rate ρ*

| 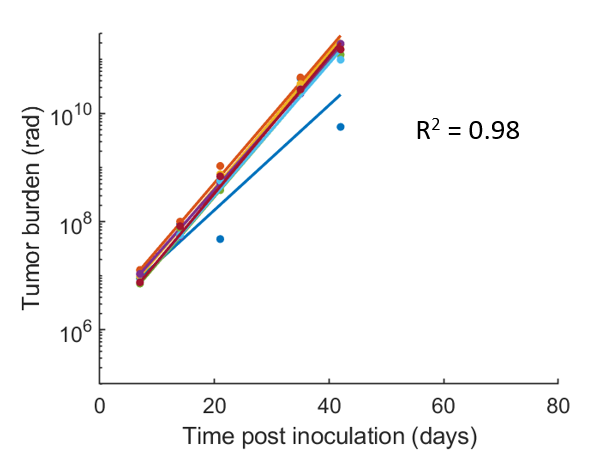 |  |  |
| --- | --- | --- |
| **Figure S1:** Estimation of tumor cell proliferation rate via exponential fits on untreated mice cohort (Group – 0) | | |

Tumor growth curves evaluated for untreated control mice showed exponential tumor growth (Figure 1). Based on the exponential fits, the proliferation rate ρ was calculated to be 0.276 ± 0.024 day^-1^.

*Single parameter set fit to all groups (global parameter set)*

The global parameter set for TRT and CAR-T cell therapies are given in Table 1. The fits for each of the mice using this parameter set is shown in figure S2A–E. The PFS and t_min_ for each of these groups as calculated from the fits is also shown where group with TRT on day 7 and CAR-T cells on day 25 results in the greatest PFS.

| 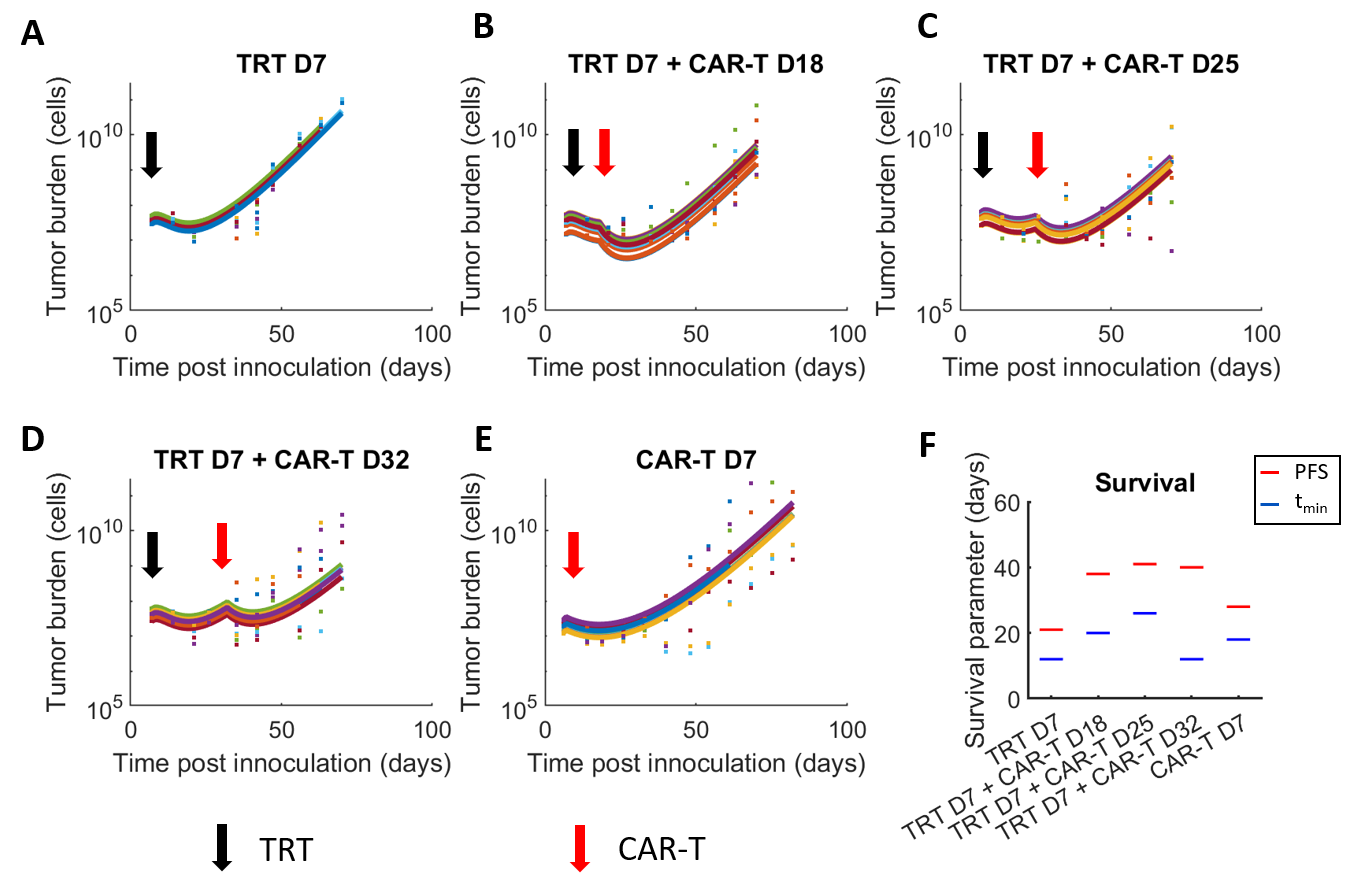 |
| --- |
| **Figure S2: Global parameter set optimization**. Optimization of single set of parameters and model fits (lines) to the tumor burden data (dots) for each treatment combination, where each color represents data from a single mouse over time. The variation in each mice data fit arises either from different initial tumor burden measured or the time of CAR-T cell therapy. **(A)** d7 TRT only **(B)** d7 TRT + d18 CAR-T cell therapy. **(C)** d7 TRT + d25 CAR-T cell therapy. **(D)** d7 TRT + d32 CAR-T cell therapy. **(E)** CAR-T cell therapy only **(F)** Survival metrics PFS and t_min_ for different groups. Each group had 8 mice except for CAR-T only group which had 7 mice. Due to the same parameter set being used for all mice there was no variability in the PFS and t_min_ observed for each mice in a cohort and all variation in tumor burden curves is accounted by differences in initial tumor burden. |

*Impact of parametric uncertainty on PFS*


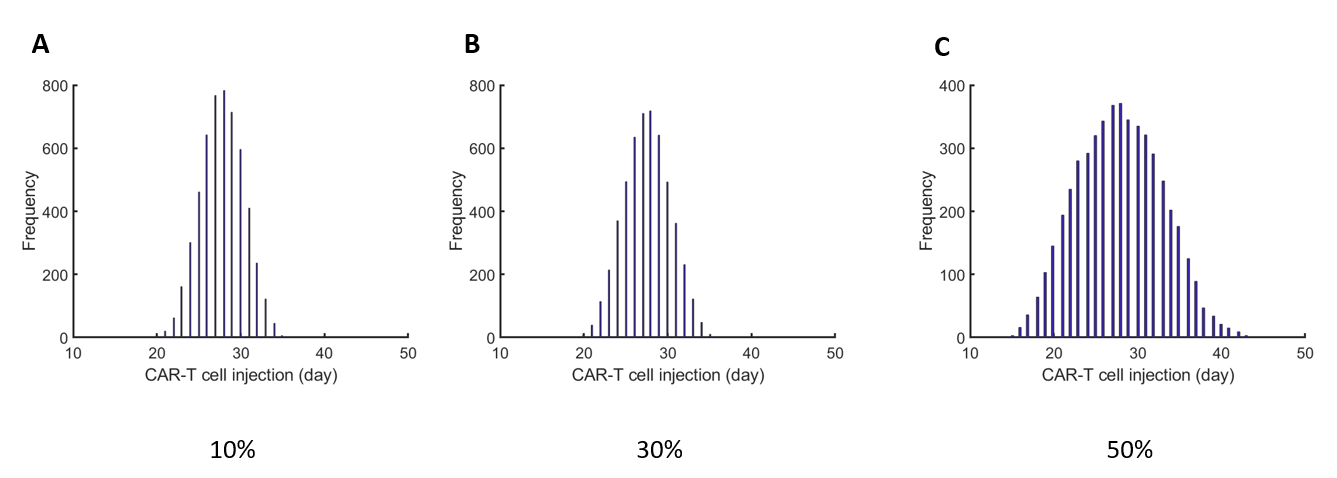


**Figure S3:** Impact of increasing uncertainty in model parameters from the global parameter set. **(A)** 10%, **(B)** 30%, **(C)** 50%. A well-defined peak at day 27-28 is seen showing it to be the day at which if CAR-T therapy is administered (after TRT on day 7), yields the highest PFS.
